# Maize/Soybean Relay Strip Intercropping Reduces the Occurrence of *Fusarium* Root Rot and Changes the Diversity of the Pathogenic *Fusarium* Species

**DOI:** 10.3390/pathogens9030211

**Published:** 2020-03-13

**Authors:** Xiaoli Chang, Li Yan, Muhammd Naeem, Muhammad Ibrahim Khaskheli, Hao Zhang, Guoshu Gong, Min Zhang, Chun Song, Wenyu Yang, Taiguo Liu, Wanquan Chen

**Affiliations:** 1 State Key Laboratory for Biology of Plant Diseases and Insect Pests, Institute of Plant Protection, Chinese Academy of Agricultural Sciences, Beijing 100193, China; xl_changkit@126.com (X.C.); zhanghao@caas.cn (H.Z.); liutaiguo@caas.cn (T.L.); 2 College of Agronomy & Sichuan Engineering Research Center for Crop Strip Intercropping system, Sichuan Agricultural University, Chengdu 611130, Sichuan Province, China; mirror_dis@126.com (L.Y.); muhammdnaeem201@gmail.com (M.N.); guoshugong@126.com (G.G.); yalanmin@126.com (M.Z.); songchun@sicau.edu.cn (C.S.); mssiyangwy@sicau.edu.cn (W.Y.); 3 Department of Plant Protection, Faculty of Crop Protection, Sindh Agriculture University, Tandojam 70060, Pakistan; mikhaskheli@sau.edu.pk; 4 National Agricultural Experimental Station for Plant Protection, Ministry of Agriculture and Rural Affairs, Tianshui 741000, Gansu Province, China

**Keywords:** Maize/soybean rely strip intercropping, soybean root rot, *Fusarium* species, population diversity, pathogenicity

## Abstract

*Fusarium* species are the most detrimental pathogens of soybean root rot worldwide, causing large loss in soybean production. Maize/soybean relay strip intercropping has significant advantages on the increase of crop yields and efficient use of agricultural resources, but its effects on the occurrence and pathogen population of soybean root rot are rarely known. In this study, root rot was investigated in the fields of the continuous maize/soybean strip relay intercropping and soybean monoculture. *Fusarium* species were isolated from diseased soybean roots and identified based on sequence analysis of *translation elongation factor 1α (EF-1α)* and *RNA polymerase* II *second largest subunit (RPB2)*, and the diversity and pathogenicity of these species were also analyzed. Our results showed that intercropping significantly decreased soybean root rot over monoculture. A more diverse *Fusarium* population including *Fusarium solani* species complex (FSSC), *F. incarnatum-equiseti* species complex (FIESC), *F. oxysporum*, *F. fujikuroi*, *F. proliferatum and F. verticillioides*, *F. graminearum* and *F. asiaticum* was identified from intercropping while FSSC, FIESC, *F. oxysporum*, *F. commune*, *F. asiaticum* and *F. meridionale* were found from monoculture. All *Fusarium* species caused soybean root infection but exhibited distinct aggressiveness. The most aggressive *F. oxysporum* was more frequently isolated in monoculture than intercropping. FSSC and FIESC were the dominant species complex and differed in their aggressiveness. Additionally, *F. fujikuroi*, *F. proliferatum and F. verticillioides* were specifically identified from intercropping with weak or middle aggressiveness. Except for *F. graminearum*, *F. meridionale* and *F. asiaticum* were firstly reported to cause soybean root rot in China. This study indicates maize/soybean relay strip intercropping can reduce soybean root rot, change the diversity and aggressiveness of *Fusarium* species, which provides an important reference for effective management of this disease.

## 1. Introduction

*Fusarium* root rot has been considered as one of the most destructive soil-borne diseases in almost all soybean growing areas worldwide and causes a drastic reduction in the optimum yield and ultimately the substantial economic losses [1]. So far, it has reported that more than 20 *Fusarium* species are pathogenic to soybean [2,3,4,5,6,7,8]. However, the diversity and pathogenicity of these *Fusarium* species can be often affected by climate factors, soybean cultivars, cropping pattern and other agricultural practices. Although high disease-resistant cultivars of soybean are still lacking [9,10], several other disease management practices including chemical fungicides [11], biological control agents [12,13], crop rotation/intercropping [14,15] and tilling [16], have been commonly adopted to control the *Fusarium* root rot.

Intercropping has been well known as one kind of the sustainable agricultural cropping patterns around the world [17,18,19]. Many studies have demonstrated that intercropping not only has obvious advantages on the increase of crop productivity [20,21,22] and efficient exploration of agricultural resources [23,24] when compared with crop monoculture, but simultaneously it can also suppress the soil-borne diseases [17]. Recent research indicated that intercropping reduced the occurrence of *Phytophthora* blight of pepper in maize/pepper intercropping [25], suppressed the incidence of *Fusarium* wilt of watermelon in rice/watermelon intercropping [26] and wheat/maize intercropping [15,27], and soybean red crown rot caused by *Cylindrocladium parasiticum* in maize/soybean intercropping [14]. Moreover, intercropping can affect soil microbial communities, reduce the attacks of pathogens and inhibit soil-borne diseases [14,17,25,28,29]. Nowadays, several intercropping patterns have been widely practiced in Asia, Latin America, Africa and other European countries regarding to local crop species and diverse climate conditions [18,19,30]. However, there is still very limited knowledge on the effects and underlying mechanisms of distinct intercropping patterns on controlling soil-borne diseases.

Recently, a maize/soybean relay strip intercropping, which is characterized by two rows of maize intercropped with two-to-four rows of soybean, has been developed in Southwest China aiming to efficiently exploit the locally limited solar-thermal and soil resources [19,31]. A range of recent studies have demonstrated that in contrast to monoculture, this creative intercropping increases crop yield [32], enhances land equivalent ratio (LER) [31], improves soil quality [33,34], suppresses field weeds [35], disease and pests [36]. Currently, the planting area of the maize/soybean relay strip intercropping system has been widely increasing in Southwest China and has also been used in the other single-season cropping areas [19]. Nevertheless, very few investigations are conducted to determine the influences of this system on soil-borne diseases such as soybean root rot.

The objectives of the present study were to investigate the incidence of soybean root rot and compare the diversity and pathogenicity of *Fusarium* species in soybean monoculture and maize/soybean relay strip intercropping. Our study will provide some references for better understanding the influence of the intercropping systems on the soil-borne diseases and the pathogen population, and this may be helpful to formulate the agricultural practices for sustainable disease management.

## 2. Results

### 2.1. Occurrence of Soybean Root Rot in Soybean Monoculture and Maize/Soybean Relay Strip Intercropping

**Figure 1:**
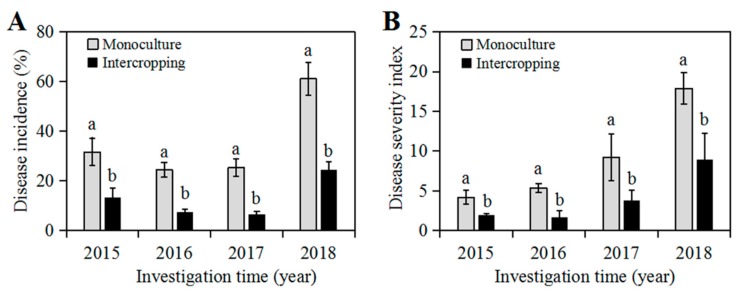
Disease incidence and disease severity index of soybean root rot in soybean monoculture and maize/soybean intercropping during 2015–2018. **A**. Disease incidence of soybean root rot; **B**. Disease severity index. Error bars indicate the standard error of the mean of three independent plots, and each is composed of 210 soybean plants. Within each year, bars with different letters are significantly different according to Duncan’s test (*p* < 0.05).

In this study, we investigated the occurrence of soybean root rot in soybean monoculture and intercropping from 2015 to 2018. We found that disease incidence (DI) of soybean root rot was steady from 2015 to 2017 in both cropping patterns, whereas a significant increase (*p* < 0.05) was observed in 2018 when the DI reached up to 61.02% in monoculture and 24.28% in intercropping, respectively (Figure 1A). In addition, the DI in monoculture was always significantly higher (*p* < 0.05) than that in intercropping each year (Figure 1A). Similarly, the disease severity index (DSI) of root rot gradually increased as the planting continued over year, and it rose up to 17.88 in monoculture in 2018 which was almost 2-fold higher than that in intercropping (Figure 1B). Thus, the continuous planting increased soybean root rot in both planting patterns, but maize/soybean relay strip intercropping was able to decrease the DI and DSI of soybean root rot as compared to monoculture.

### 2.2. Identification of Fusarium Species from Soybean Monoculture and Maize/Soybean Relay Strip Intercropping

After isolation and purification, a total of 37 isolates were obtained from diseased soybean roots in monoculture when compared with 36 isolates in intercropping (Table 1). Partial sequences of *translation elongation factor 1α* (*EF-1α*) and *RNA polymerase* II *second largest subunit* (*RPB2*) gene were sequenced and blasted against the databases of *Fusarium MLST* (http://www.wi.knaw.nl/Fusarium/Biolomics.aspx) and FUSARIUM-ID (http://isolate.fusariumdb.org/guide.php) to identify *Fusarium* species. Our results showed that these isolates had more than 98% sequence similarity with 10 species or species complex including *Fusarium solani* species complex (FSSC), *Fusarium incarnatum-equiseti* species complex (FIESC), *F. oxysporum*, *F. commune,* three species (*F. fujikuroi*, *F. proliferatum, F. verticillioides)* belonging to *Fusarium fujikuroi* species complex (FFSC) and three species (*F. graminearum, F. asiaticum* and *F. meridionale*) in the *Fusarium graminearum* species complex (FGSC) (Table 1).

**Figure 2:**
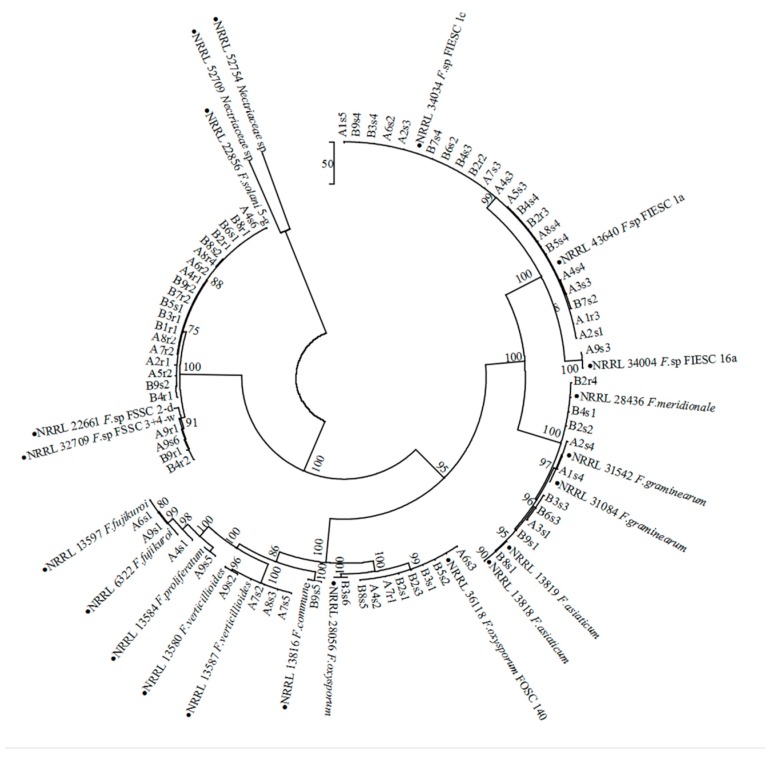
Phylogenetic tree of *Fusarium* species isolated from soybean monoculture and intercropping based on both *EF-1α* and *RPB2* gene. The maximum-parsimony (MP) tree was constructed based on both *EF-1α* and *RPB2* genes with MEGA6.0. Sequences of *Nectriaceae* sp. (NRRL52709 and NRRL52754) were selected to root the maximum parsimony phylogeny as an outgroup. Numbers at internodes represent MP bootstrap support based on 1000 replicates. Reference sequences of *Fusarium* isolates from GenBank are indicated by a solid circle.

For phylogenetic analysis, the maximum-parsimony (MP) tree was constructed based on both *EF-1α* and *RPB2* gene sequences of 37 isolates from monoculture, 36 isolates from intercropping, 22 referred isolates and 2 *Nectriaceae* sp. isolates (NRRL52709 and NRRL52754) as outgroup in Figure 2. The parameters for this tree were followed as 1053 for tree length (TL), 0.762 for consistency index (CI), 0.975 for retention index (RI) and 0.734 for rescale consistency index (RCI), respectively. As shown in Figure 2, all isolates were clearly classified into 10 species or species complex including FIESC, FSSC, *F. oxysporum*, *F. commune, F. fujikuroi*, *F. proliferatum, F. verticillioides, F. graminearum, F. asiaticum* and *F. meridionale*, which was in agreement with Blastn analysis. The clade bootstrap support values were over 96 for all species and species complex branches. In addition, there was no obvious intraspecies genetic differentiation of *Fusarium* population between monoculture and intercropping. All sequences of *EF-1a* and *RPB2* genes have been submitted to NCBI database and GenBank accession numbers were listed in Table 1.

### 2.3. Diversity and Isolation Frequency of Fusarium Species from Monoculture and Intercropping

**Figure 3:**
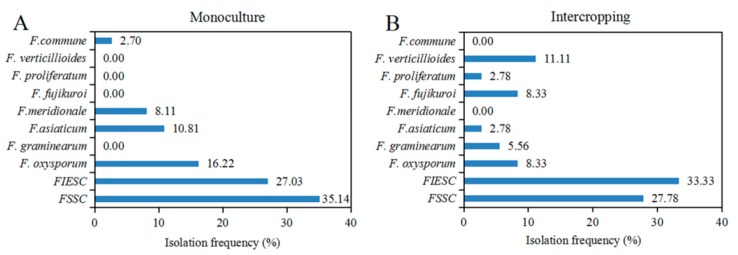
Isolation frequency of *Fusarium* species isolated from soybean monoculture and intercropping. The values on the bar graph stand for the isolation frequency, which is calculated using the percentage of the isolates of each *Fusarium* species in total isolates obtained from either monoculture (**A**) or intercropping (**B**).

As shown in Figure 3, there was a significant difference (*p* = 0.038, Fisher’s exact test) in the isolation frequency of *Fusarium* species, and a higher diversity was observed in intercropping than monoculture at species level. FSSC and FIESC had the higher isolation frequency than other species in both cropping patterns. About 8.33% of *F. oxysporum* was obtained in intercropping as compared to 16.22% in soybean monoculture. *Fusarium graminearum* and *F. asiaticum* belonging to *FGSC* covered 8.34% of those isolates in intercropping, whereas a combination of *F. asiaticum* and *F. meridionale* in the same species complex accounted for 18.92% in monoculture. We also noticed that *F. verticillioides, F. proliferatum* and *F. fujikuroi* in the FFSC were specifically isolated from intercropping pattern with the isolation frequency for 11.11%, 2.78% and 8.33%, respectively, while *F. commune* as the specific species exhibited 2.7% of *Fusarium* isolates from monoculture. Thus, our results demonstrated that *FSSC* and FIESC were the dominant species in both cropping systems, but the specifically-isolated FFSC coupled with almost 2-fold lower percentage of both *F. oxysporum* and species in the FGSC was clearly present in maize/soybean relay strip intercropping rather than soybean monoculture.

### 2.4. Pathogenicity of Fusarium Species from Monoculture and Intercropping

**Figure 4:**
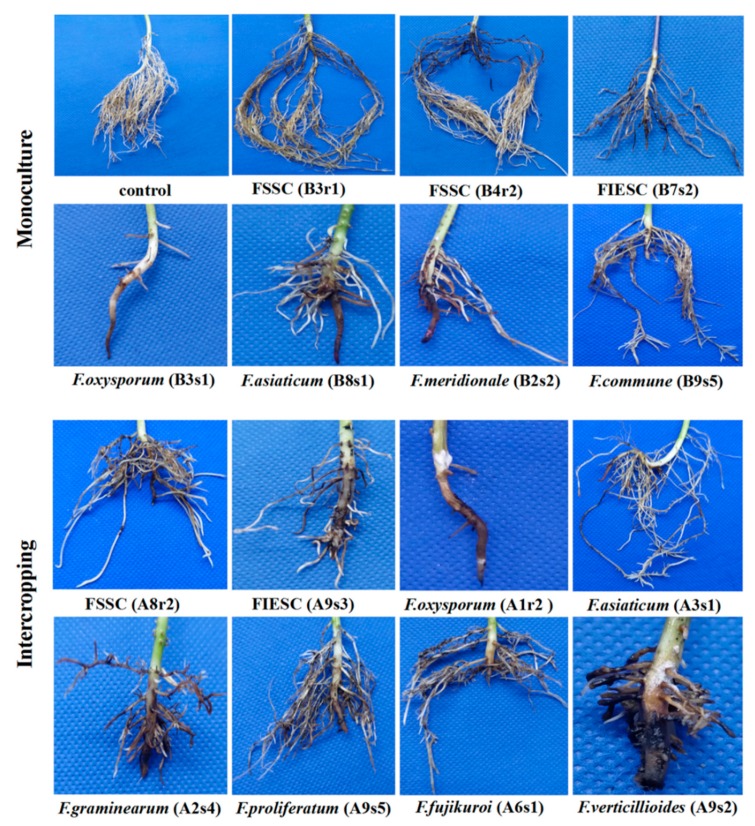
Pathogenicity test of *Fusarium* species on soybean cultivar Nandou12. Pathogenicity test was performed using sorghum grain infected by the representative *Fusarium* isolates. FSSC (B3r1, B4r2), FIESC (B7s2), *F. oxysporum* (B3s1), *F. asiaticum* (B8s1), *F. meridionale* (B2s2) and *F. commune* (B9s5) were isolated from monoculture, while *FSSC* (A8r2), FIESC (A9s3), *F. oxysporum* (A1r2), *F. asiaticum* (A3s1), *F. graminearum* (A2s4), *F. proliferatum* (A9s5), *F. fujikuroi* (A6s1) and *F. verticillioides* (A8s3) were isolated from intercropping. Control stand for the soybean without *Fusarium* inoculation.

**Figure 5:**
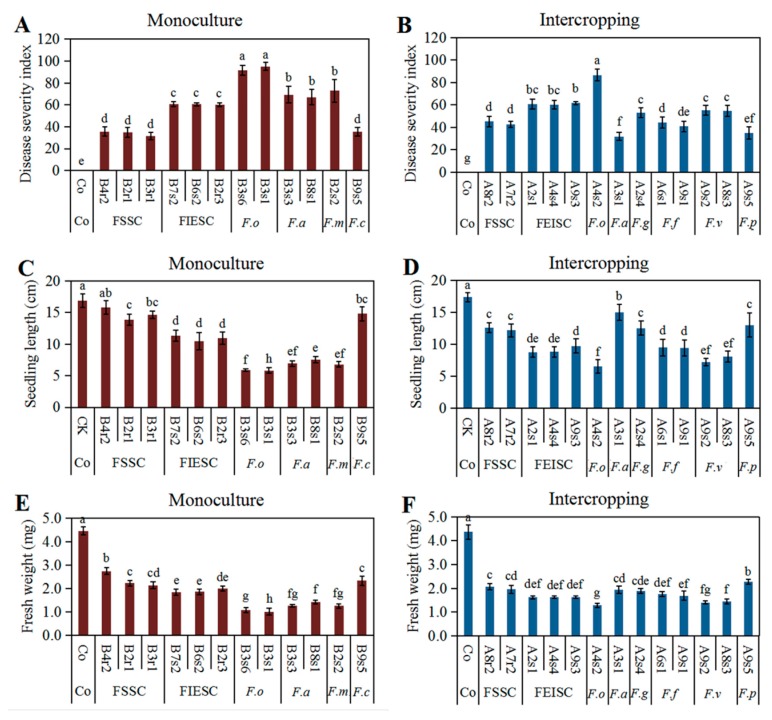
Growth parameters and disease severity index of soybean after inoculation with *Fusarium* species from monoculture and intercropping. The means of disease severity index, seedling length and fresh weight were obtained from three independent experiments after inoculated with the representative isolates of *Fusarium* species from monoculture (**A**, **C**, **E**) and intercropping (**B**, **D**, **F**). Co, the seedlings without *Fusarium* inoculation used as control; FSSC, *Fusarium solani* species complex; FIESC, *Fusarium incarnatum-equiseti* species complex; *F.o, F. oxysporum*; *F.a*, *F. asiaticum*; *F.m*, *F.meridionale*; *F.c*, *F.commune*; *F.g*, *F.graminearum*; *F.f*, *F.fujikuroi*, *F.v*, *F.verticillioides*; *F.p*, *F.proliferatum*. Error bars indicate standard error of the mean of three replicates, and each is composed of 18 plants. Bars with different letters are significantly different according to Duncan’s test (*p* > 0.05) using SPSS 21 software.

To test the pathogenicity of *Fusarium* species identified from soybean monoculture and maize/soybean relay strip intercropping, symptoms of soybean root rot were observed after 15-day inoculation with the representative *Fusarium* isolates, and the disease severity index (DSI) was calculated according to disease severity caused by *Fusarium* species. Our results showed that all the representative isolates were able to infect soybean root and caused stunted, brown, rotted taproots and hair roots when compared with the control soybean seedlings (Figure 4). There was obvious different aggressiveness for each *Fusarium* species, but the representative isolates of each *Fusarium* species had almost the same DSI in the corresponding cropping pattern. In addition, *Fusarium* infection resulted in a significant reduction of seedling length and fresh weight of soybean compared with the un-inoculated control soybean in both monoculture (Figure 5A,C) and intercropping (Figure 5C,D).

Among all *Fusarium* species, *F. oxysporum* was the most aggressive species with the DSI up to 91.59 in monoculture and 86.63 in intercropping, respectively (Figure 5A,B), and meanwhile it sharply reduced seedling height and fresh weight (Figure 5C–F), affected the development of hair root and resulted in severe rotted taproots of soybean seedlings (Figure 4). Regarding the species belonging to FGSC, *F. meridionale* (B2s2) and *F. asiaticum* (B3s3 and B8s1) from monoculture were secondly aggressive to soybean seedlings and caused obvious rotted taproots while *F. graminearum* (A2s4) and *F. asiaticum* (A3s1) from intercropping had relatively lower DSI. Although both FIESC and FSSC were most frequently isolated in both cropping patterns, they showed different aggressiveness when infected the soybean seedlings. FIESC caused nearly the same disease symptoms in both cropping patterns, whereas FSSC from intercropping was more aggressive than those from monoculture (Figure 5A,B). Interestingly, *F. fujikuroi*, *F. verticillioides* and *F. proliferatum* in the FFSC which were specially identified from intercropping had obvious difference in their aggressiveness after inoculation on soybean with the DSI ranging from 34.92 (A9s5) to 54.50 (A8s3) (Figure 5). Moreover, *F. commune* (B9s5) as one specific species of monoculture had weak aggressiveness on soybean. In general, our results demonstrated that there were more aggressive *Fusarium* species with high isolation frequency in monoculture than intercropping, and meanwhile these *Fusarium* species had also much stronger inhibition effects on seedling length and fresh weight. 

## 3. Discussion

**Figure 6:**
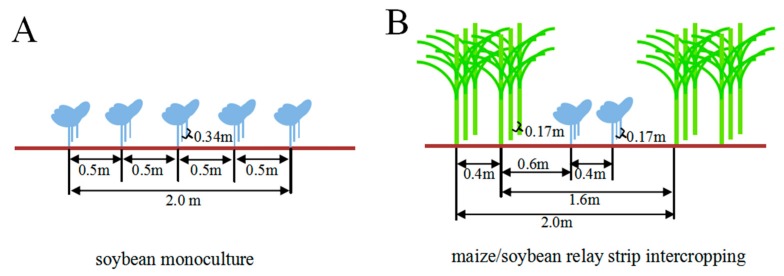
The planting pattern of location field experiments for soybean monoculture and maize/soybean strip intercropping. **A**. Soybean monoculture; **B**. Maize/soybean relay strip intercropping.

Intercropping has been widely used as an effective agricultural strategy to control soil-borne diseases [17]. Previous studies have proved that intercropping reduces *Phytophthora* blight of pepper [25], *Fusarium* wilt of watermelon [15,27], and red crown rot of soybean [14]. Consistently, our studies found that the maize/soybean relay strip intercropping significantly decreased the incidence of soybean root rot by approximately 18.5%–36.7% compared with monoculture (Figure 1). A recent report has revealed that maize plant forms an unavailable non-host “root wall” when intercropped with pepper, thus decreasing the *Phytophthora* disease of pepper [25]. Under our maize/soybean intercropping pattern, roots of two-row maize plants constitute a physical barrier between two-row soybean strips below the ground (Figure 6B), and this special root space distribution might be very difficult for the dispersion of pathogenic *Fusarium* species across soybean roots in the rhizosphere [43]. In addition, some reports demonstrated that root secretion from intercropped wheat inhibited spore germination, sporulation and growth of the soil-borne *F. oxysporum* f. sp. *niveum* causing *Fusarium* wilt of watermelon [15], and on the other hand it also enhanced the crop resistance through the induction of *PRs* gene expression and accumulation of phenolic acids in a root-dependent manner [14,15]. Regarding to our intercropping system, maize plants were relay strip intercropped with soybean with the distance of 0.6 cm that probably is difficult for direct root interaction, but this specific intercropping contributes a lot to a positive interspecific facilitation, improves root nutrition and increases productivity of soybean crop [34,44]. Therefore, we predict that intercropped maize might alter the pathogen establishment and enhance the soybean resistance through formation of root barrier or root interaction.

Previous studies have demonstrated that negative interactions among *Fusarium* species through competition for space niche and feed resources can effectively affect *Fusarium* growth and their infection on hosts [45,46]. It has showed that *F. verticillioides* has a better growth and a higher spore production than *F. graminearum* when co-inoculated with maize [45]. Other studies found that as the most important pathogenicity factor of *Fusarium* species, mycotoxin production was also significantly changed in competing interactions. For example, fumonisins, mainly produced by *F. verticillioides*, *F. proliferatum* and *F. fujikuroi,* were commonly reduced in competing interactions, whereas the DON produced by *F. graminearum* was increased [47]. In this study, we identified a total of 10 *Fusarium* species/complex including FIESC, FSSC, *F. oxysporum, F. verticillioides*, *F. fujikuroi*, *F. proliferatum*, *F. commune*, *F. graminearum*, *F. asiaticum* and *F. meridionale* in location field experiments from two cropping patterns, which is nearly consistent with our previous studies that a co-occurring *Fusarium* species caused soybean root rot in this region [48]. We also observed a more diverse *Fusarium* population (8/10) in intercropping than those (6/10) in monoculture, but the DSI over 60.0% caused by *Fusarium* species accounted for 25.0% (2/8) in intercropping which was lower than 66.7% (4/6) in monoculture. This indicated that low disease incidence and disease severity index of soybean root rot in intercropping might be associated with a more diverse *Fusarium* community and their negative interactions, thus resulting in low pathogenic responses to soybean.

To be worthwhile, *F. vericillioides, F. fujikuroi* and *F. proliferatum* classified into the FFSC were specifically isolated from intercropping. Actually, they are among important pathogens causing maize ear rot and stalk rot in Sichuan Province and other countries [49,50,51,52]. In this study, the co-growth period for maize and soybean lasts more than two months in our maize/soybean relay strip intercropping pattern, and this might provide enough time for the dispersion of *Fusarium* spores causing maize stalk rot to soybean becoming the inoculums of seedling blight and pod decay of soybean [53]. Furthermore, after harvest, these *Fusarium* species also can survive saprophytically and accumulate largely on maize and soybean debris, even on or inside field soil, thus may serve as primary inocula for soybean infection in the following year [54]. From this point, there is the possibility for intercropping to increase the risks of root rot through the cyclic infection of some *Fusarium* species in maize and soybean. Thus, it is very necessary to conduct field studies to further investigate the cross-pathogenicity and cyclic infection of these common species, and how they could be managed in an integrated system by effective agricultural practices.

Moreover, we identified three members of FGSC including *F. graminearum*, *F. asiaticum* and *F. meridionale* as the pathogens of soybean root rot in this location field experiment, in particular, *F. asiaticum* and *F. meridionale* were firstly reported from soybean in China. It is well-known that *F. graminearum* is the most destructive pathogens of *Fusarium* head blight (FHB) [55,56] and maize ear and stalk rot in China and many other countries [51,52,57,58], while *F. asiaticum* was more frequently isolated with isolation frequency up to 60% than *F. graminearum* in Southwest China [56]. *Fusarium meridionale* is the predominant species on maize in Nepal and Northern Argentina [56] but at quite low frequency in other countries such as South Korea [59]. In our previous studies, *F. meridionale* was only rarely isolated from wheat and maize in Southwest China including Sichuan and its surrounding areas [56,57], but the factors which influence its distribution are still unknown. In this study, we found that *F. meridionale* was specifically associated with monoculture system, indicating that *F. meridionale* may show host preference on soybean rather than maize and wheat. Some researches suggested that the cropping system was more important in determining the regional prevalence of *Fusarium* species associated with FHB [60,61]. *Fusarium graminearum* is more frequently associated with maize and wheat rotation when *F. asiaticum* appear in rice and wheat rotation in Korea [61]. Similarly, multiple intercropping patterns such as rice and maize rotation, wheat/maize and maize/soybean intercropping are widely practiced in Sichuan Province of China [19], and this could put a selective stress on different hosts and help these species of FGSC to shift onto soybean from wheat or maize [56,59]. In addition, we observed that the disease incidence of root rot significantly increased in 2018 as compared to the previous years in two cropping patterns, which was followed by more rainfall days, especially mid of July 2018 during the soybean growth stage (Figure S1). This indicates some certain positive correlation exists between the incidence of *Fusarium* root rot and rainfall. However, the disease incidence was much higher in monoculture than intercropping, implying that intercropping has much stronger tolerance to unfavorable rainfall. Some studies have demonstrated that unusual climate changes such as severe drought and rainfall damage the natural capacity of soil microbes to suppress soil-borne plant pathogens, contributing to increased disease outbreaks [62].

Currently, the maize/soybean relay strip intercropping has been recognized by Chinese government as one major promotional agricultural cropping pattern because of its competitive advantages on the high yield per unit area and the increase of land equivalent rations (LER) over the traditional maize/soybean intercropping and soybean monoculture [31,35,63]. Our previous studies have demonstrated that the total crop yield of the maize/soybean relay strip intercropping (RI_wn_) was significantly higher than the traditional maize/soybean intercropping with (RI_e_) under a normal growing year, and the average LER of the grain yield was 1.79 for RI_wn_ and 1.49 for RI_e_, respectively [63]. When compared with soybean monoculture, this specific intercropping decreased soybean yield to a small extent based on a 2-year field experiment, but additive relay strip intercropped maize increased the total crop yield in the intercropping system [63]. In this current study, a 7-year continuous intercropping caused 24.8% lower disease incidence of soybean root rot than monoculture, but the final yield of soybean was not evaluated in both cropping patterns. In the further work, the relationship between disease incidence and the crop yield under maize/soybean relay strip intercropping will be further analyzed to deep understand the underlying mechanism of this intercropping on soybean root rot and *Fusarium* community.

## 4. Materials and Methods

### 4.1. Field Experiments

The continuous 7-year location-specific field experiments of the maize/soybean rely strip intercropping and soybean monoculture without tillage were conducted at Yucheng District, Yaan City, P. R. China (29°59″3.17′N, 102°59″2.57′E). The soil in this field was observed as a purple clay texture with pH 6.71 and 2.86% organic matter, and the available N, P and K in the top soil profile (0–30 cm) of 117 mg·kg^−1^, 31.3 mg·kg^−1^ and 68.2 mg·kg^−1^, respectively. The randomized complete block design was used for both intercropping and monoculture with three replicated experimental plots. Each plot was designed for 36 m^2^ with 6 m in width and 6 m in length, comprising 3 strip units in intercropping and 5 rows of soybean in monoculture. For monoculture, the distance of soybean row was 0.5 m and the hole space was 34 cm in Figure 6A. The maize/soybean relay strip intercropping was planted as shown in Figure 6B according to Yang et al. [30]. For intercropping, two-row maize was spaced with two-row soybean in 2 m width as one strip intercropping unit, where the row space was 0.4 m for maize-maize or soybean-soybean and 0.6 m for maize-soybean, and the hole space was 17 cm for both crops. The soybean plants were about 210 in each field of both planting systems. Maize cultivar “Denghai605” and soybean cultivar “Nandou12” were continuously used from 2012 to 2018. Maize was sown in late May and harvested in Mid-August, and soybean was sown in Mid-June and harvested in late October, respectively. All these experiment field were not tilled every year before sowing.

### 4.2. Investigation and Sampling of Soybean Root Rot

Soybean root rot was surveyed from maize/soybean relay strip intercropping and soybean monoculture at R_2_ growth stages according to Killebrew et al. [64] from 2015 to 2018. The disease incidence (DI) and disease severity index (DSI) of root rot were calculated from three replicated experimental plots according to the formula referred to Liu et al. [12]. Total of 18 plants, showing typical symptoms of brown taproots and hair roots, withered and yellowish leaves, were collected from each location field in two planting patterns and were used for pathogen isolation.

### 4.3. Isolation of Fusarium Species

The diseased roots were washed with tap water, cut into small pieces (approximately 1 mm^2^), and surface-sterilized with 75% ethanol (v/v) for 30 s, 1% NaOCl (w/v) for 10 s and rinsed three times with sterile distilled water. These pieces were placed on Petri dishes containing potato dextrose agar (PDA, 200 g∙L^−1^ potato, 10 g∙L^−1^ glucosum anhydricum and 15 g∙L^−1^ agar), and then incubated at 25 °C for 7–10 days in the darkness. All isolates were purified by cutting marginal hyphae and then transferred onto new PDA dishes as test isolates for further analysis according to Chang et al. [48]. The information of isolates was listed in Table 1.

### 4.4. PCR Amplification and Phylogenetic Analysis

For molecular identification, the fungal mycelia were collected from 7-day-old isolates on PDA dishes and then grounded in liquid nitrogen with a disposable pellet pestle. Total genomic DNA of all isolates was extracted using a SP Fungal DNA Kit (Aidlab Biotech, Chengdu, China) according to the manufacturer’s protocols. Partial gene sequences of *translation elongation factor 1-alpha* (*EF-1α*) and *RNA polymerase beta large subunit II* (*RPB2*) were amplified using the primer pairs EF1/EF2 [38] and RPB2-5f2 /RPB2-7cr [38], respectively. PCR reaction was conducted in a total volume of 25 μL containing DNA template 1 μL, each primer 1 μL (10 μM), Taq PCR Mastermix (Sangon Biotech, Shanghai, China) 12.5 μL and DNase free water 9.5 μL. Amplification conditions were 5 min at 94 °C, followed by 35 cycles of denaturation at 94 °C for 30 s, annealing at 55 °C for 30 s, initial extension at 72 °C for 1 min and kept at 72 °C for 10 min. PCR products were detected by 1.5% agarose gel electrophoresis and then sequenced by an ABI-PRISM3730 automatic sequencer (Applied Biosystems, Foster, CA, USA) in Sangon Biotech Co., Ltd. (Shanghai, China).

For phylogenetic analysis, amplified sequences of *EF-1α and RPB2* gene were blasted on the databases of FUSARIUM-ID (http://isolate.fusariumdb.org/guide.php) and *Fusarium MLST* (http://www.wi.knaw.nl/Fusarium/Biolomics.aspx). The referred isolate information of *Fusarium* species were obtained and listed in Supplementary Materials Table S1. All sequences were edited and aligned with Clustal X 1.83, and characters were weighed equally. A maximum-parsimony (MP) tree was conducted with MEGA 6.0 using the Nearest-Neighbor-Interchange Heuristic method based on both *EF-1α* and *RPB2* sequences. Clade support was inferred from 1000 bootstrap replicates, and alignment gaps were excluded. The tree parameters including tree length (TL), consistency index (CI), retention index (RI) and rescale consistency index (RCI) were also calculated. Novel sequence data were deposited in GenBank and the alignment in TreeBASE (www.treebase.org).

### 4.5. Pathogenicity Tests of Fusarium Species

To complete Koch’s postulates, the representative strains from all identified *Fusarium* species were tested for pathogenicity on the seedling of soybean cultivar Nandou12 through inoculated sorghum grains as described by Chang et al. [48]. Sorghum grains were soaked in distilled water in a 250 mL flask overnight and autoclaved for 60 min twice. About 10–15 pieces of mycelium plugs from PDA colonies after 5 days culture were inoculated into sorghum grains and incubated at 25 °C in the dark for 10 days to obtain the infested sorghum grains. After germination, seeds of cv. Nandou 12 were sowed in plastic pots containing the mixture of autoclaved Pindstrup substrate with infested sorghum grains (3:1, w/w). Soybean seeds were un-inoculated with sorghum grains as control. Nine plastic pots for each isolate were prepared with two seeds per pot. All inoculated soybeans were incubated in a chamber at 25 °C with 85% relative humidity under 12 h light alternated with 12 h darkness. This experiment was performed for 3 independent replicates. After 15 days of inoculation, soybean seedlings were removed from the substrate and washed using running tap water. The presence of root rot symptoms and disease severity were evaluated using 0–4 scale as previously described by Chang et al. [48] with some modifications as follows: 0 = no symptoms, 1 = mild symptoms (discoloration but no visible lesions), 2 = obvious lesions (severe discoloration with lateral root reduction), 3 = severe lesions on the primary and lateral root and diminished plant vigor and 4 = stem rotten, plant dead. Disease severity index (DSI) was calculated according to the formula as follows. In addition, the seedling height and fresh weights of soybean were recorded.

(1) $$DSI=\frac{\sum \left(severityrating\times seedlingperrating\right)}{\left(totalseedlings\times highestseverityrating\right)}\times 100$$

### 4.6. Statistic Analysis of Data

All data were recorded and basically processed using Microsoft office Excel. Mean values of disease incidence (DI) and disease severity index (DSI) in disease field investigation were calculated from three experimental plots of soybean monoculture and maize/soybean relay strip intercropping. In the pathogenicity test, mean values of DSI, seedling height and fresh weight of soybean plants after inoculation with the representative *Fusarium* isolates were obtained from three independent replicates with 18 seedlings each treatment. The data correlation was conducted by generalized linear model (GLM) with quasipoisson distribution for residuals, and statistical analysis was conducted by Duncan’s test with GLM function using IBM SPSS Statistics 21. Significance difference was set at the level of *p* = 0.05. The isolation frequency was calculated using the percentage of the isolates of each *Fusarium* species in total isolates from either monoculture or intercropping, and difference of isolation of *Fusarium* species in each cropping pattern was analyzed by Fisher’s exact test.

## 5. Conclusions

In this study, the effects of maize/soybean relay strip intercropping on soybean root rot, the diversity and pathogenicity of *Fusarium* species were investigated in a continuous location field experiment when compared with soybean monoculture. We found that this typical intercropping significantly reduced soybean root rot and disease severity as compared to monoculture. Meanwhile, *Fusarium* species causing soybean root rot in intercropping were more diverse and less aggressive than those in monoculture. Thus, it can be concluded that maize/soybean relay strip intercropping has some positive effects on the reduction of *Fusarium* root rot probably because of the diversity increase of *Fusarium* species coupled with their low pathogenicity as a whole. However, the underlying mechanisms on the interaction of soybean root and soil microbes in the rhizosphere need to be further explored. In addition, in this study, several *Fusarium* species identified from soybean are also the pathogens of maize and pre-crop wheat, probably leading to the inoculum accumulation and cross-pathogenicity among crops, and this will increase the risk for the optimum productivity of intercropping. With respect to this, the cross-pathogenicity mechanism of the predominant *Fusarium* species on intercrops should be elucidated, which will provide a valuable reference for the integrated management of *Fusarium* root rot in soybean.

## Figures and Tables

**Table 1:** Information of *Fusarium* isolates obtained from soybean monoculture and intercropping and GenBank accession numbers of *EF-1α* and *RPB2*.

Isolate Code	Planting Pattern	GenBank Accession Number		*Fusarium* Species ^a^	*Fusarium* Species Complex
		*EF-* *1* *α*	*RPB2*		
A1s4	Intercropping	MK560306	MN892318	*F. graminearum*	*Fusarium graminearum* species complex, FGSC [37]
A1s5	Intercropping	MK560320	MN892327	FIESC	*Fusarium incarnatum-equiseti* species complex, FIESC [38,39]
A1r3	Intercropping	MK560319	MN892328	FIESC	FIESC
A2s1	Intercropping	MK560321	MN892326	FIESC	FIESC
A2s3	Intercropping	MK560322	MN892325	FIESC	FIESC
A2s4	Intercropping	MK560307	MN892317	*F. graminearum*	FGSC
A2r1	Intercropping	MK560280	MN892289	FSSC	*Fusarium solani* species complex, FSSC [40]
A3s1	Intercropping	MK560335	MN892344	*F. asiaticum*	FGSC
A3s3	Intercropping	MN892351	MN892338	FSSC	FSSC
A4s1	Intercropping	MK560308	MN892321	*F. fujikuroi*	*Fusarium fujikuroi* species complex, FFSC [41]
A4s2	Intercropping	MK560300	MN892307	*F. oxysporum*	*Fusarium oxysporum* species complex, FOSC [8,39,40]
A4s3	Intercropping	MK560323	MN892336	FIESC	FIESC
A4s4	Intercropping	MK560324	MN892341	FIESC	FIESC
A4s6	Intercropping	MK560283	MN892295	*F. solani*	FSSC
A4r1	Intercropping	MK560282	MN892296	FSSC	FSSC
A5s3	Intercropping	MK560325	MN892342	FIESC	FIESC
A5r2	Intercropping	MK560284	MN892284	FSSC	FSSC
A6s1	Intercropping	MK560309	MN892320	*F. fujikuroi*	FFSC
A6s2	Intercropping	MK560326	MN892324	FIESC	FIESC
A6s3	Intercropping	MK560301	MN892306	*F. oxysporum*	FOSC
A6r2	Intercropping	MK560285	MN892294	*F. solani*	FSSC
A7s2	Intercropping	MK560261	MN892281	*F. verticillioides*	FFSC
A7s3	Intercropping	MK560327	MN892323	FIESC	FIESC
A7s5	Intercropping	MK560262	MN892280	*F. verticillioides*	FFSC
A7r1	Intercropping	MK560302	MN892305	*F. oxysporum*	FOSC
A7r2	Intercropping	MK560286	MN892293	*F. solani*	FSSC
A8s3	Intercropping	MK560263	MN892279	*F. verticillioides*	FFSC
A8s4	Intercropping	MK560328	MN892343	FIESC	FIESC
A8r2	Intercropping	MK560288	MN892291	FSSC	FSSC
A8r4	Intercropping	MK560287	MN892292	*F. solani*	FSSC
A9s1	Intercropping	MK560310	MN892319	*F. fujikuroi*	FFSC
A9s2	Intercropping	MK560264	MN892278	*F. verticillioides*	FFSC
A9s3	Intercropping	MK560329	MN892322	FIESC	FIESC
A9s5	Intercropping	MK560292	MN892349	*F. proliferatum*	FFSC
A9s6	Intercropping	MK560290	MN892285	FSSC	FSSC
A9r1	Intercropping	MK560291	MN892290	FSSC	FSSC
B1r1	Monoculture	MK560265	MN892304	FSSC	FSSC
B2s1	Monoculture	MK560293	MN892313	*F. oxysporum*	FOSC
B2s2	Monoculture	MK560303	MN892316	*F. meridionale*	FGSC
B2s3	Monoculture	MK560294	MN892312	*F. oxysporum*	FOSC
B2r1	Monoculture	MK560266	MN892288	FSSC	FSSC
B2r2	Monoculture	MK560311	MN892335	FIESC	FIESC
B2r3	Monoculture	MK560312	MN892337	FIESC	FIESC
B2r4	Monoculture	MK560304	MN892315	*F. meridionale*	FGSC
B3s1	Monoculture	MK560295	MN892311	FIESC	FOSC
B3s3	Monoculture	MK560331	MN892348	*F. asiaticum*	FGSC
B3s4	Monoculture	MK560313	MN892334	FIESC	FIESC
B3s6	Monoculture	MK560296	MN892310	*F. oxysporum*	FOSC
B3r1	Monoculture	MK560267	MN892303	FSSC	FSSC
B4s1	Monoculture	MK560305	MN892314	*F. meridionale*	FGSC
B4s3	Monoculture	MK560314	MN892333	FIESC	FIESC
B4s4	Monoculture	MK560315	MN892339	FIESC	FIESC
B4r1	Monoculture	MK560268	MN892283	FSSC	FSSC
B4r2	Monoculture	MK560269	MN892286	FSSC	FSSC
B5s1	Monoculture	MK560270	MN892302	FSSC	FSSC
B5s2	Monoculture	MK560297	MN892309	*F. oxysporum*	FOSC
B5s4	Monoculture	MN892352	MN892332	FSSC	FSSC
B6s1	Monoculture	MK560272	MN892301	FSSC	FSSC
B6s2	Monoculture	MK560316	MN892331	FIESC	FIESC
B6s3	Monoculture	MK560332	MN892347	*F. asiaticum*	FGSC
B7s2	Monoculture	MK560317	MN892340	FIESC	FIESC
B7s4	Monoculture	MK560318	MN892330	FIESC	FIESC
B7r2	Monoculture	MK560273	MN892300	FSSC	FSSC
B8s1	Monoculture	MK560333	MN892346	*F. asiaticum*	FGSC
B8s2	Monoculture	MK560274	MN892299	FSSC	FSSC
B8s4	Monoculture	MN892354	MN892308	FSSC	FSSC
B8r1	Monoculture	MK560276	MN892298	FSSC	FSSC
B9s1	Monoculture	MK560334	MN892345	*F. asiaticum*	FGSC
B9s2	Monoculture	MK560277	MN892282	FSSC	FSSC
B9s4	Monoculture	MN892353	MN892329	FIESC	FIESC
B9s5	Monoculture	MK560330	MN892350	*F. commune*	*Fusarium nisikadoi* species complex, FNSC [42]
B9r1	Monoculture	MK560278	MN892287	FSSC	FSSC
B9r2	Monoculture	MK560279	MN892297	FSSC	FSSC

Note: ^a^ indicate that *Fusarium* species were identified based on the phylogenetic analysis of both *translation elongation factor 1α* (*EF-1α*) and *RNA polymerase II second largest subunit* (*RPB2*) genes on *Fusarium MLST* and FUSARIUM-ID database.

## Supplementary Materials

The following are available online at https://www.mdpi.com/2076-0817/9/3/211/s1, Figure S1: Investigation of rainfall days at the growth stage of soybean during 2015–2018 in Ya’an City. Table S1: Information of reference isolates and GenBank accession numbers of *RPB2* and *EF-1α* used for the phylogenetic analysis of *Fusarium* species causing soybean root rot isolated from soybean monoculture and maize/soybean relay strip intercropping.
