# Supplementary material for: Maize/Soybean Relay Strip Intercropping Reduces the Occurrence of Fusarium Root Rot and Changes the Diversity of the Pathogenic Fusarium Species
Source: Pathogens. 2020 Mar 13;9(3):211. doi: 10.3390/pathogens9030211 (PMC7157700; doi:10.3390/pathogens9030211)
Supplement: Supplementary file 1 [file pathogens-09-00211-s001.pdf]

Supplementary material

Maize/Soybean Relay Strip Intercropping Reduces the Occurrence of *Fusarium* Root Rot and Changes the Diversity of the Pathogenic *Fusarium* Species

Xiaoli Chang [1,2,†], Li Yan [2,†], Muhammd Naeem [2,†], Muhammad Ibrahim Khaskheli [3], Hao Zhang [1], Guoshu Gong [2], Min Zhang [2], Chun Song [2], Wenyu Yang [2], Taiguo Liu [1,4] and Wanquan Chen [1,4,*]

1 State Key Laboratory for Biology of Plant Diseases and Insect Pests, Institute of Plant Protection, Chinese Academy of Agricultural Sciences, Beijing 100193, China; xl_changkit@126.com (X.C.); zhanghao@caas.cn (H.Z.); liutaiguo@caas.cn (T.L.)

2 College of Agronomy & Sichuan Engineering Research Center for Crop Strip Intercropping system, Sichuan Agricultural University, Chengdu 611130, Sichuan Province, China; mirror_dis@126.com (L.Y.); muhammdnaeem201@gmail.com (M.N.); guoshugong@126.com (G.G.); yalanmin@126.com (M.Z.); songchun@sicau.edu.cn (C.S.); mssiyangwy@sicau.edu.cn (W.Y.)

3 Department of Plant Protection, Faculty of Crop Protection, Sindh Agriculture University, Tandojam 70060, Pakistan; mikhaskheli@sau.edu.pk

4 National Agricultural Experimental Station for Plant Protection, Ministry of Agriculture and Rural Affairs, Tianshui 741000, Gansu Province, China

* Correspondence: wqchen@ippcaas.cn; Tel: +86-10-62815618, Fax: +86-10-62895365

† These authors contributed equally to this work.

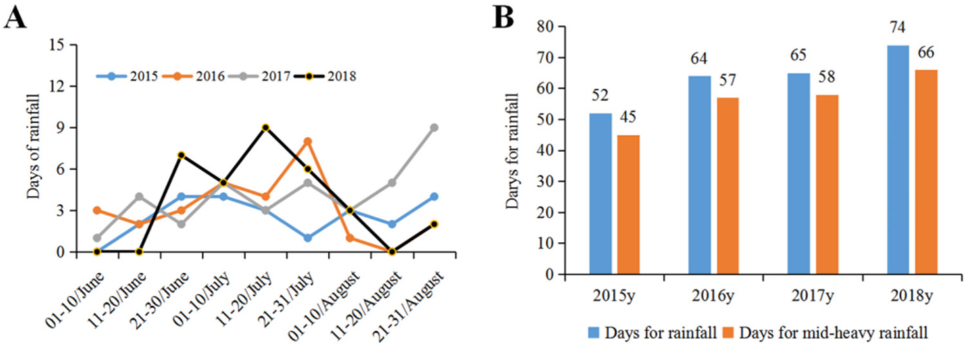

Figure S1 Investigation of rainfall days at the growth stage of soybean during 2015–2018 in Ya'an City. The field location experiments of the maize/soybean relay strip intercropping and soybean monoculture were conducted at Yucheng District, Yaan City, China. The rainfall at the growth stage of soybean during 2015–2018 in Ya'an City were investigated from July 1st to August 31th during 2015–2018.

Table S1 Information of reference isolates and Genbank accession numbers of *RPB2* and *EF-1α* used for the phylogenetic analysis of *Fusarium* species causing soybean root rot isolated from soybean monoculture and maize/soybean relay strip intercropping.

Isolate code	GenBank accession number		*Fusarium* species
	EF-1α	***RPB2***	
NRRL 34034 1-c	GQ505636	GQ505814	FIESC
NRRL 43640 1-a	GQ505667	GQ505845	FIESC
NRRL 34004	GQ505628	GQ505806	FIESC
NRRL 28436	AF212435	KM361660	*F. meridionale*
NRRL 31542	MH582236	MH582101	*F. graminearum*
NRRL 31084	HM744693	JX171644	*F. graminearum*
NRRL 13819	MH582250	MH582121	*F. asiaticum*
NRRL 13818	MH582249	MH582120	*F. asiaticum*
NRRL 36118	FJ985330	LS479221	*F. oxysporum*
NRRL 28056	MH582353	MH582140	*F. oxysporum*
NRRL 13816	MH582348	MH582181	*F. commune*
NRRL 13587	MH582328	MH582189	*F. verticillioides*
NRRL 13580	MH582323	MH582214	*F. vericillioides*
NRRL 13584	MH582346	MH582172	*F. proliferatum*
NRRL 6322	MH582338	MH582103	*F. fujikuroi*
NRRL 13597	MH582339	MH582156	*F. fujikuroi*
NRRL 32709 3+4-w	DQ247029	FJ240397	FSSC
NRRL 22661 2-d	DQ246846	EU329524	FSSC
NRRL 22856 5-g	MH582423	MH582228	*F. solani*
NRRL 52709	JF740791	JF741117	*Nectriaceae sp.*
NRRL 52754	JF740829	JF741155	*Nectriaceae sp.*

Note: *RPB2, RNA polymerase II second largest subunit*; *EF-1α, the translation elongation factor 1-α.*
